# Enhanced performance of flexible quantum dot light-emitting diodes using a low-temperature processed PTAA hole transport layer

**DOI:** 10.1038/s41598-023-30428-y

**Published:** 2023-03-07

**Authors:** Hyoun Ji Ha, Min Gye Kim, Jin Hyun Ma, Jun Hyung Jeong, Min Ho Park, Seong Jae Kang, Wonsik Kim, Soohyung Park, Seong Jun Kang

**Affiliations:** 1 Department of Advanced Materials Engineering for Information and Electronics, Kyung Hee University, Yongin, 17104 Republic of Korea; 2 Integrated Education Program for Frontier Materials (BK21 Four), Kyung Hee University, Yongin, 17104 Republic of Korea; 3 Advanced Analysis Center, Korea Institute of Science and Technology, 5 Hwarang-Ro 14-Gil, Seongbuk-Gu, Seoul, 02792 Republic of Korea; 4 Division of Nano & Information Technology, KIST School, University of Science and Technology (UST), Seoul, 02792 Republic of Korea

**Keywords:** Materials science, Optics and photonics

## Abstract

Low-temperature processing is important for improving the stability and performance of flexible quantum dot light-emitting diodes (QLEDs). In this study, QLEDs were fabricated using poly[bis(4-phenyl) (2,4,6-trimethylphenyl)amine] (PTAA) as a suitable hole transport layer (HTL) material owing to its low-temperature processability and vanadium oxide as the low-temperature solution-processable hole injection layer material. The maximum luminance and highest current efficiency of the QLEDs on a glass substrate with an optimal PTAA HTL was 8.9 × 10^4^ Cd/m^2^ and 15.9 Cd/A, respectively, which was comparable to that of conventional devices. The QLEDs on a flexible substrate showed a maximum luminance of 5.4 × 10^4^ Cd/m^2^ and highest current efficiency of 5.1 Cd/A. X-ray and ultraviolet photoelectron spectroscopies were used to investigate the chemical state and interfacial electronic structure according to the materials and the state changes of the HTL, respectively. The interfacial electronic structure showed that PTAA exhibited a better hole transport ability owing to its low hole injection barrier (${\Phi }_{{\text{h}}}$). Moreover, QLEDs with a PTAA HTL could operate as photosensors under reverse bias conditions. These results indicate that the low-temperature-processed PTAA HTL is suitable for improving the performance of flexible QLEDs.

## Introduction

Flexible displays have recently been highlighted as they can be applied to mobile and wearable devices^1^. However, flexible displays have problems such as difficulty in realizing color, high driving voltage, and thin-film separation. To overcome the problems of flexible displays, high-performance light-emitting diodes (LEDs) using various light-emitting materials, such as quantum dots (QDs), perovskite, and organic materials, are being studied. In particular, QD LEDs (QLEDs) have the advantages of excellent color purity, narrow emission spectrum bandwidth, high luminance, and low production cost; therefore, intensive research is required^2–4^.

Plastic substrates, such as polyethylene terephthalate and polyethylene naphthalate (PEN), are used for flexible QLEDs because of their flexibility, strong impact resistance, convenient processing, and light weight^5,6^. However, thermal deformation of the plastic substrate is induced in the high-temperature process, leading to a decrease in the QLEDs performance^7^. To solve this problem, materials that can be processed at low temperatures and substrates with high thermal stability can be used, and many studies have been conducted on both. Because the thermal stability of plastic substrates is limited, a material that can be processed at low temperatures is particularly important; therefore, research on a material capable of exhibiting excellent performance even at a low temperatures is required.

Rigid QLEDs commonly use poly[(9,9-dioctylfluorenyl-2,7-diyl)-co-(4,4'-(4-s-butylphenyl)diphenylamine)] (TFB) as the hole transport layer (HTL) owing to its high hole mobility^8,9^. However, the TFB HTL requires a high-temperature process of approximately 180 °C, which can affect the substrate. Therefore, it is necessary to find HTL materials for flexible QLEDs that can be processed at low temperatures to replace TFB. Poly[bis(4-phenyl) (2,4,6-trimethylphenyl)amine] (PTAA) is a completely amorphous and π-conjugated polymer that forms uniform thin films and enables appropriate charge transport^10^. Furthermore, PTAA has low-temperature processability (below 100 °C), which prevents deformation of the plastic substrate^11,12^. Therefore, PTAA was applied as a suitable HTL material to enhance the performance of QLEDs.

Additionally, because QLEDs and QD photodiodes (PDs) have the same device structure, two functions can be performed in one device. In the forward bias, the device acts as an LEDs, and in the reverse bias, the device acts as a PD. Therefore, it can be further developed and utilized for biosensors and optical communication using visible-light emission and sensing characteristics by adjusting the bias.

In this study, QLEDs were fabricated using a PTAA HTL on a flexible substrate. The device was optimized based on the concentration of PTAA and the UV-ozone (UVO) treatment time. An appropriate UVO treatment time increased the hydrophilicity and conductivity of the PTAA layer, improving the performance of the QLEDs. X-ray photoelectron spectroscopy (XPS) was performed to investigate the chemical changes in PTAA after UVO treatment. Ultraviolet photoelectron spectroscopy (UPS) was used to confirm the improvement in the energy-band alignment of the hole-injection part according to the HTL change. A maximum luminance, current efficiency, and external quantum efficiency (EQE) of 8.9 × 10^4^ Cd/m^2^, 15.9 Cd/A, and 3.5%, respectively, demonstrated the improved performance of the rigid QLEDs. The respective values attained for the flexible QLEDs were 5.4 × 10^4^ Cd/m^2^, 5.1 Cd/A, and 1.1%. Additionally, the photodetection abilities of the PTAA device were confirmed. This study demonstrates that the PTAA HTL is suitable for flexible QLEDs and has the potential for application in bidirectional optical signal transmission.

## Results and discussion

**Figure 1:**
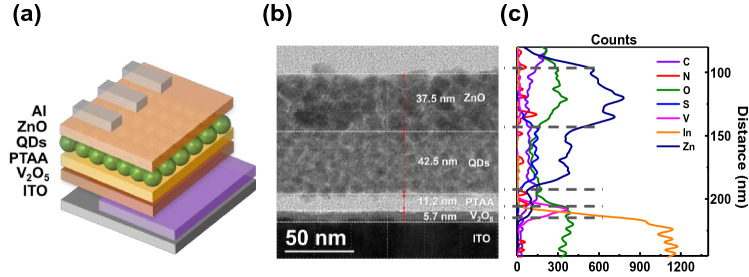
(**a**) Schematic structure of the QLEDs with UVO 40. (**b**) Cross-sectional HR-TEM image of the QLEDs with UVO 40. (**c**) EDS line scan of the elemental distribution.

Figure 1a shows a schematic structure of the QLEDs with the PTAA HTL. Figure 1b shows a cross-sectional HR-TEM image of the QLEDs with UVO 40, which demonstrates that layers of V_2_O_5_/PTAA/green QDs/ZnO were deposited on the glass/ITO. The thicknesses of each layer were 5.7, 11.2, 42.5, and 37.5 nm, respectively. Figure 1c shows a quantitative comparison of the number of elements according to the EDS line-scan results. Figure S1 shows EDS mapping images of indium, vanadium, carbon, and selenium, which indicate that the deposited layers did not diffuse into the underlayer. This is important for high-performance devices requiring uniform and clear interfacial thin films^13^.

**Figure 2:**
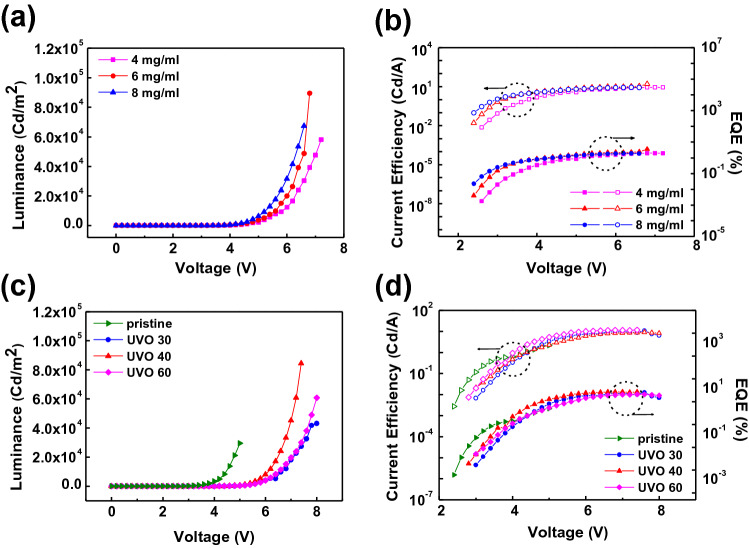
Performance of QLEDs with PTAA HTLs at various concentrations. (**a**) L-V and (**b**) CE-V-EQE curves. Performance of QLEDs with PTAA HTLs with various UVO times. (**c**) L-V and (**d**) CE-V-EQE curves. The QLEDs were fabricated on an ITO glass substrate.

Figure 2 shows the performance of the QLEDs with the PTAA HTL under various conditions. Table 1 summarizes the performances of the QLEDs on the glass/ITO substrate. Figure 2a,b shows the luminance-voltage (L-V) and current efficiency–voltage-EQE (CE-V-EQE) characteristics of the QLEDs with 4, 6, and 8 mg/ml PTAA, respectively. At 6 mg/ml, the maximum luminance, current efficiency, and EQE were 8.9 × 10^4^ Cd/m^2^, 15.9 Cd/A, and 3.5%, respectively, showing the highest performance. QLEDs performance at 2, 4, 6, 8 and 8 mg/ml PTAA concentrations for optimization is summarized in Table S1. Figure 2c,d show the L-V and CE-V-EQE characteristics of QLEDs with pristine PTAA, UVO 30, UVO 40, and UVO 60. At UVO 40, the maximum luminance, current efficiency, and EQE were 8.4 × 10^4^ Cd/m^2^, 11.6 Cd/A, and 2.7%, respectively, showing the highest performance. The performances of QLEDs with respect to the UVO treatment time are summarized in Table S2. These results showed that the optimal conditions for QLEDs fabrication were 6 mg/ml and UVO 40.

**Table 1:** Device performance of the QLEDs on the ITO glass substrates with TFB and various PTAA HTLs.

Device	${\text{L}}_{{{\text{max}}}}$ (Cd/${\text{m}}^{{2}}$)	Turn on V (V)	${\text{CE}}_{{{\text{max}}}}$ (Cd/A)	${\text{PE}}_{{{\text{max}}}}$ (Im/W)	EQE (%)	Full width half maximum (nm)	EL ${\uplambda }_{{{\text{max}}}}$ (nm)
4 mg/ml	8.5 × 10^4^	2.9	8.8	3.6	1.9	24.9	538
6 mg/ml	8.9 × 10^4^	2.7	15.9	7.3	3.5	24.6	535
8 mg/ml	6.7 × 10^4^	2.7	8.5	4.0	1.9	24.8	537
UVO 30	4.3 × 10^4^	3.3	6.4	2.5	1.5	23.9	532
UVO 40	8.4 × 10^4^	3.3	11.6	4.9	2.7	24.1	533
UVO 60	6.1 × 10^4^	3.3	8.3	3.2	1.9	24.2	533
PTAA pristine	2.9 × 10^4^	2.3	2.2	1.4	0.5	24.1	537
TFB	7.5 × 10^4^	3.3	11.0	4.3	2.5	23.4	534

**Figure 3:**
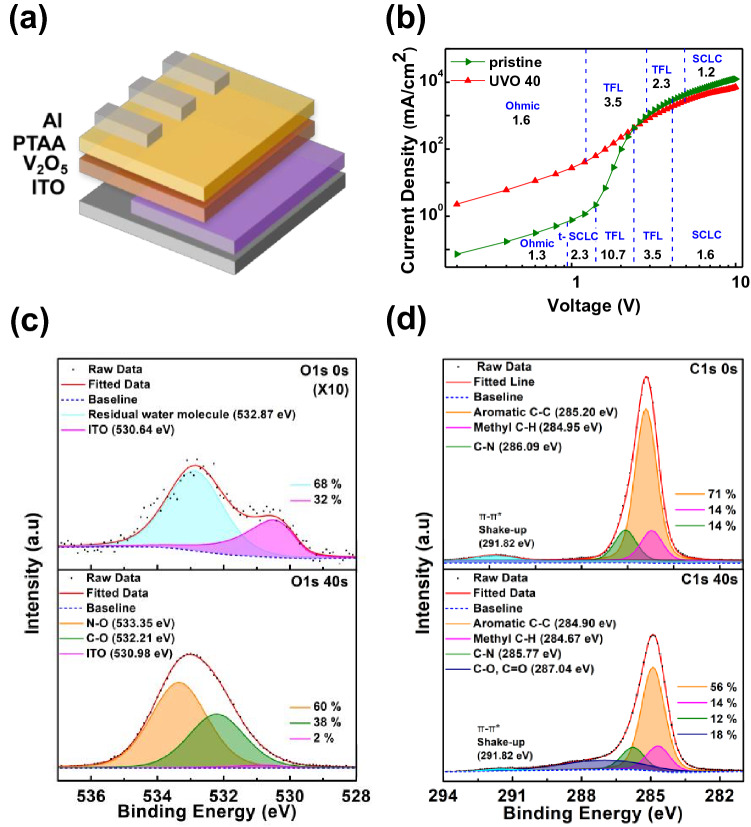
(**a**) Schematic structure of the HOD with PTAA HTL. (**b**) J-V curves of the pristine HOD and HOD with UVO 40. (**c**) O 1 s and (**d**) C 1 s XPS spectra of the pristine and UVO 40 PTAA films on ITO.

To investigate the origin of the enhanced hole-transport capacity through UVO treatment, a hole-only device (HOD) was fabricated. Figure 3a shows the schematic structure of the HOD with PTAA. Figure 3b shows a comparison of the current density–voltage (J-V) characteristics of the HOD with and without UVO treatment on the PTAA layer. In a pristine device, there are four regions, namely, the ohmic contact, Child’s law of space charge limited currents (SCLC), trap partially-filled SCLC (t-SCLC), and traps-filled-limited (TFL) regions^14^. However, there was no t-SCLC region in UVO 40 and the TFL current was lower than that without UVO treatment. This means that the UVO-treated PTAA layer had enhanced hole mobility and a lower trap concentration, as compared to that of the pristine PTAA layer^15^. As shown in Figure S2, the hole mobility improved with an increasing UVO treatment time until 40 s. However, the UVO treatment time of 60 s excessively destroyed the polymer chain structure of PTAA, and the device performance degraded.

XPS measurements were performed to determine the origin of the improvement in hole mobility by confirming the chemical state change of the PTAA layer after UVO treatment. Figure 3c,d show the XPS core-level spectra of oxygen (O 1 s) and carbon (C 1 s), respectively. The spectra of pristine PTAA are shown in the upper part of each figure, and those of UVO 40 are shown in the lower part. Figure S3a shows that the ITO oxygen peak appeared at 530.66 eV. Therefore, the oxygen peaks for each PTAA film found at 530.64 eV and 530.98 eV were due to the underlying ITO substrate. In pristine PTAA, the residual water molecule corresponds to 532.87 eV^16^. After UVO treatment, N–O and C–O bonds were additionally observed at 533.35 and 532.21 eV with the disappearance of residual water molecules. Additionally, the XPS core-level spectra of nitrogen (N 1 s) are shown in Figure S3b and c. In pristine PTAA, only N–C bonds were observed at 400.12 eV, and the UVO treatment reduced the proportion of N–C bonds at 399.81 eV and formed a N–O bond at 400.62 eV. This indicates that the UVO treatment broke the part of polymer bond (~ 18%) and recombined it with oxygen. This was also observed for the C 1 s peak of the PTAA film. The pristine PTAA film exhibited three C 1 s peaks, which corresponded to the aromatic C–C, methyl C–H and C–N bonds at 285.20, 284.95, and 286.09 eV, respectively. The UVO treatment broke the first three bonds at 284.90, 284.67, and 285.77 eV and C–O or C = O bonds were newly formed at 287.04 eV. The increase in the number of oxygen bonds indicates that the PTAA interface became hydrophilic. This can also be confirmed by measuring the contact angles (CA). As shown in Figure S4a and c, the CA of the PTAA interface decreased from 81.86 to 50.39° after the UVO treatment for 40 s. Figure S4b and d shows the CAs for each UVO treatment time. The enhanced hydrophilicity of the PTAA surface made the HTL deposition more uniform and smoother. Additionally, the XPS results showed that the UVO treatment oxidized the PTAA surface. As the hole concentration on the oxidized PTAA surface increases, the conductivity improves, which can enhance the performance of the QLEDs^17^.

**Figure 4:**
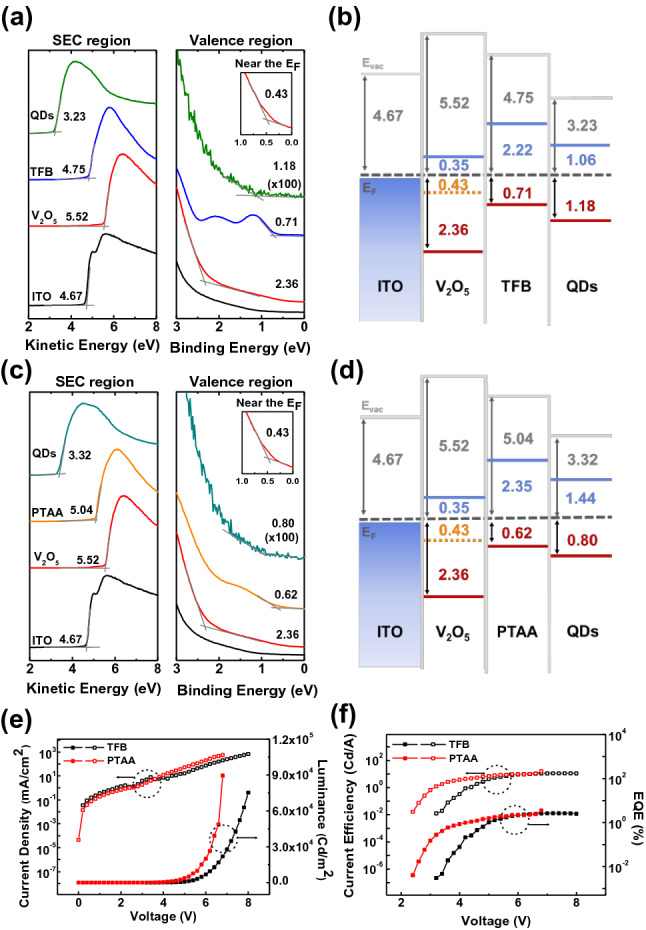
UPS spectra at the SEC, valence, and near the E_F_ regions. (**a**) ITO, ITO/V_2_O_5_, ITO/V_2_O_5_/TFB, and ITO/V_2_O_5_/TFB/QDs. (**c**) ITO, ITO/V_2_O_5_, ITO/V_2_O_5_/PTAA (UVO 40), and ITO/V_2_O_5_/PTAA (UVO 40)/QDs. Energy level diagram of (**b**) ITO/V_2_O_5_/TFB/QDs and (**d**) ITO/V_2_O_5_/PTAA (UVO 40)/QDs. Performance of the QLEDs with UVO 40 and TFB HTLs. (**e**) J-V-L and (**f**) CE-V-EQE curves.

UPS was used to investigate the interfacial electronic structure of the hole-transport region of the QLEDs using each HTL. Figure 4a,b show the secondary electron cutoff (SEC) and valence region spectra of ITO, ITO/V_2_O_5_, ITO/V_2_O_5_/TFB, ITO/V_2_O_5_/PTAA (UVO 40), ITO/V_2_O_5_/TFB/QDs, and ITO V_2_O_5_/PTAA (UVO 40)/QDs. The WFs of ITO and V_2_O_5_ were 4.67 ($\pm$ 0.005 eV) and 5.52 eV ($\pm$ 0.005 eV), respectively, as shown in the SEC region. The valence band maximum (VBM) of V_2_O_5_ in the valence region was 2.36 eV below E_F_. Additionally, the insets of Fig. 4a and c show the gap state in V_2_O_5_ at 0.43 eV was observed near the E_F_. The gap state in V_2_O_5_, which plays a valuable role in the injection of hole carriers, appeared with the density of state between VBM and E_F_^18^. As shown in Fig. 4a, the WF of the TFB and QDs were 4.75 ($\pm$ 0.005 eV) and 3.23 eV ($\pm$ 0.005 eV), respectively, and the highest occupied energy state molecular orbital (HOMO) of TFB and VBM of the QDs were 0.71 and 1.18 eV, respectively. Figure 4c shows that the WFs of the PTAA and QDs were 5.04 ($\pm$ 0.005 eV) and 3.32 eV ($\pm$ 0.005 eV), respectively, and that the HOMO of PTAA and VBM of the QDs were 0.62 and 0.80 eV, respectively. Figure 4b and d show the energy level diagrams of the hole transport region with TFB and PTAA HTL as obtained from the UPS spectra. The conduction band minimum and lowest unoccupied molecular orbital of each layer were calculated as the optical energy band gap (E_g_), VBM, and HOMO values. The E_g_ of each layer was calculated using Tauc’s plots of the absorbance spectra measured using UV–vis spectroscopy, as shown in Figure S5. The hole injection barrier (${\Phi }_{{\text{h}}}$) at the V_2_O_5_/PTAA interface was 0.19 eV, which was 0.09 eV lower than that at the V_2_O_5_/TFB interface. Additionally, when compared with the ${\Phi }_{{\text{h}}}$ of the HTL/emission material layer (EML) interface, the ${\Phi }_{{\text{h}}}$ of the PTAA/QD interface was 0.18 eV, which was 0.29 eV lower than the ${\Phi }_{{\text{h}}}$ of the TFB/QD interface of 0.47 eV. A lower ${\Phi }_{{\text{h}}}$ at each interface leads to an improved device performance. The HOD J-V characteristics shown in Figure S6 demonstrates that the PTAA HTL exhibited better conductivity, as the J values of the TFB and PTAA devices were 469.81 and 863.91 mA/cm^2^, respectively, at 3 V. The number of injected hole carriers and the J value were associated with the ${\Phi }_{{\text{h}}}$ values, with a lower ${\Phi }_{{\text{h}}}$ increasing the J value^19^. Additionally, Fig. 4e and f show the current–voltage-luminance (J-V-L) and CE-EQE characteristics of the QLEDs with each HTL. The maximum luminance, current efficiency, and EQE of the TFB device were 7.5 × 10^4^ Cd/m^2^, 11.0 Cd/A, and 2.5%, respectively, which were lower than those of the optimized PTAA device (6 mg/ml and UVO 40). The luminance of the device at the same voltage was higher in the PTAA device than in the TFB device with a higher J value after 3.6 V. These results show that by reducing ${\Phi }_{{\text{h}}}$, the PTAA HTL provides better energy level alignment and the QLEDs correspondingly achieve a better performance than when using the TFB HTL.

**Figure 5:**
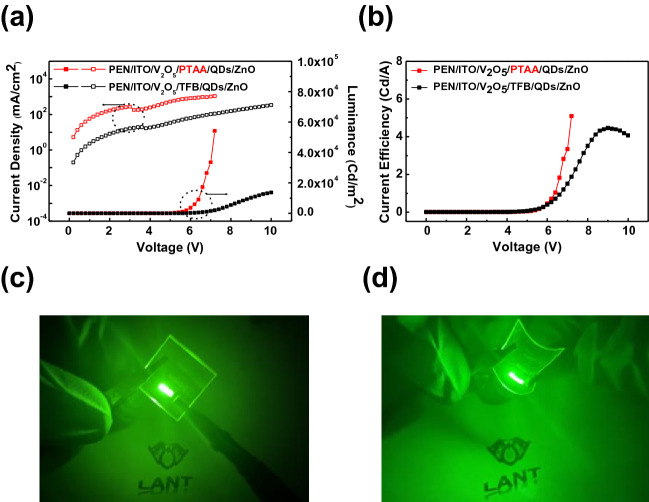
Performance of the QLEDs on the PEN substrate with PTAA and TFB HTLs. (**a**) J-V-L and (**b**) CE-V curves. (**c** and **d**) Photographs of the flexible QLEDs.

Flexible QLEDs were fabricated on a PEN substrate using a TFB and a PTAA HTL. The PTAA device was prepared using UVO 40. Figure 5a,b show the J-V-L and CE-V characteristics of the QLEDs. Table 2 summarizes the performances of the QLEDs on the PEN/ITO substrate. The J value of the PTAA device was tenfold higher than that of the TFB devices at the same voltage. Additionally, the luminance of the PTAA device was significantly higher than that of the TFB device. The maximum luminance of the PTAA and TFB devices was 5.4 × 10^4^ and 1.4 × 10^4^ Cd/m^2^, respectively. As the sheet resistance of the PEN/ITO substrate, as compared to that of the glass/ITO substrate, increased from 10–11 to 15 Ω/sq, the device performance decreased as the substrate was changed to a flexible one. Additionally, annealing at 180 °C for 30 min increased the sheet resistance and substrate deformation curvature of PEN/ITO^7^. The overall device performance of PTAA on the flexible substrate was significantly better than that on TFB. Therefore, it was demonstrated that device performance degradation was induced by the thermal deformation of the PEN substrate due to the high temperature. This also indicates that low-temperature processed PTAA is suitable for flexible QLEDs. Figure 5c,d show photographs of the flat and bent flexible PTAA devices.

**Table 2:** Device performance of the QLEDs on the PEN substrates with TFB and PTAA HTLs.

Device	${\text{L}}_{{{\text{max}}}}$ (Cd/${\text{m}}^{{2}}$)	Turn on V (V)	${\text{CE}}_{{{\text{max}}}}$ (Cd/A)	${\text{PE}}_{{{\text{max}}}}$ (Im/W)	EQE (%)	Full width half maximum (nm)	EL ${\uplambda }_{{{\text{max}}}}$ (nm)
UVO 40	5.4 × 10^4^	3.5	5.1	2.2	1.1	27.5	542
TFB	1.4 × 10^4^	4.1	4.1	1.3	0.9	23.9	533

**Figure 6:**
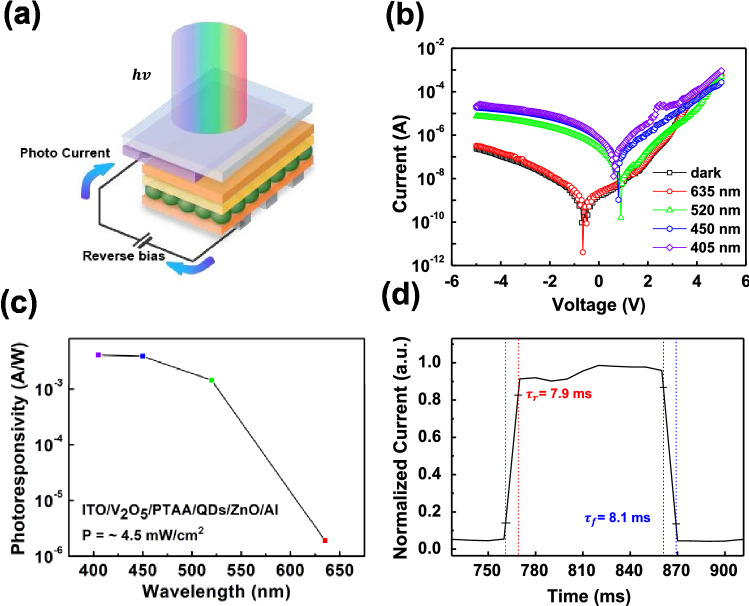
(**a**) Schematic structure of the PDs. (**b**) I-V of the device in the dark state and under various light wavelengths. (**c**) Photoresponsivity of the PD at 635, 520, 450, and 405 nm light wavelengths. (**d**) Photo response characteristics of PD. The rising and falling times were defined at the interval between 10 and 90% of the signal.

Furthermore, the PTAA device can be used as a PD under reverse bias conditions. Figure 6a shows a schematic diagram of the glass substrate. Figure 6b shows the I-V curve of the device under dark conditions and light illumination with 405, 450, 520, and 635 nm wavelengths. It was difficult to observe the photocurrent in the 635 nm wavelength because its photon energy (1.95 eV) was not sufficient to excite green QD carriers with an E_g_ of 2.24 eV. The photon energies of the remaining wavelengths were 2.38, 2.75, and 3.06 eV, respectively, which were sufficient to operate the device as a photodetector. The photocurrent was represented by the photoresponsivity calculated using the following equation:

$${\text{Photoresponsivity}} = \frac{{I_{l} - I_{d} }}{{{\text{PA}}}}$$

where ${\text{I}}_{{\text{l}}}$ is the current under various wavelengths of light, ${\text{I}}_{{\text{d}}}$ is the dark current, *A* is the illuminated area of the device, and P is the power intensity of the laser source. The P of each laser source was kept to be 4.5 mW/cm^2^. Figure 6c shows the calculated photoresponsivity. The photoresponsivity of the PTAA device significantly increased under light illumination at a wavelength of 520 nm. Figure 6d shows the rising ($\tau_{r}$) and falling times ($\tau_{f}$), defined as the time to reach 10 and 90% of the maximum normalized current value, respectively, under 520 nm wavelength light illumination. The $\tau_{r}$ and $\tau_{f}$ were 7.9 and 8.1 ms, respectively. The PTAA device was operated as a QLEDs and a PD by adjusting the bias. Therefore, PTAA can be used to apply low-temperature processes in QLEDs and PD manufacturing. Furthermore, this technology could be key to future solution-processing optics manufacturing.

## Conclusions

Flexible QLEDs were fabricated in this study using a PTAA HTL via low-temperature solution-processing. The PTAA layer was treated with UVO to increase hydrophilicity and conductivity. The UVO effect was confirmed by measuring the XPS spectra and current–voltage characteristics of the HOD. UPS measurements confirmed the alignment of the energy levels between the HIL/HTL and HTL/EML interfaces. It was found that the ${\Phi }_{{\text{h}}}$ of each interface was reduced by 0.09 and 0.29 eV, respectively. The interfacial electronic structure shows that QLEDs with PTAA have better hole transport characteristics. The enhanced hole transfer ability was observed using the J-V curve of the HOD. The QLEDs with the PTAA HTL showed a maximum luminance of 8.9 × 10^4^ Cd/m^2^ and a current efficiency of 15.9 Cd/A when the device was fabricated on a glass substrate. The luminous performance of the flexible QLEDs with the PTAA HTL was 5.4 × 10^4^ Cd/m^2^, which was more than five times higher than that of the control device. QLEDs with PTAA can be operated as photosensors under reverse bias conditions. Therefore, our device can be used as a photosensor and LEDs. This result shows that the PTAA HTL is suitable for flexible QLEDs owing to its low-temperature processability and has potential for application in optical communication systems.

## Methods

### Synthesis of vanadium oxide (V_2_O_5_)_,_ TFB, and PTAA solutions

A V_2_O_5_ solution (1 wt%) was synthesized by adding 0.05 ml vanadium triisopropoxide oxide to 7 ml isopropyl alcohol. 0.1 ml deionized water was added to activate the hydrolysis reaction. The resulting solution was stirred for 30 min. A TFB (1 wt%) solution was prepared by mixing 0.0173 g of TFB into 2 ml p-xylene and stirring for 30 min at 600 rpm. PTAA solutions of various concentrations (2, 4, 6, 8 and 10 mg/ml) were synthesized by dissolving PTAA powders in chlorobenzene and stirring overnight at room temperature.

### QLED fabrication

Patterned indium tin oxide (ITO) glass and PEN substrates were ultrasonically cleaned with DI water, acetone, and isopropyl alcohol for 15 min each. The work function (WF) and hydrophilicity of the ITO was increased using a 15 min UVO treatment. The V_2_O_5_ HIL was spin-coated onto the ITO anode at 3000 rpm for 60 s and annealed at 25 °C for 25 min. The HTL, TFB, and PTAA were deposited by spin-coating at 3000 and 4000 rpm for 30 s. They were then annealed at 180 °C for 30 min and 100 °C for 10 min. This was then followed by a UVO treatment on the PTAA surface for 0 (pristine), 30 (UVO 30), 40 (UVO 40), 60 (UVO 60) and 180 s (UVO 180). The CdSe/ZnS QD solution was spin-coated at 2000 rpm for 30 s onto each HTL and annealed at 90 °C for 10 min. The ZnO electron transport layer was spin-coated at 2000 rpm for 60 s and annealed at 90 °C for 10 min. An 130 nm-thick aluminum cathode was deposited to complete the device fabrication using a thermal evaporator.

### Characterization

The device structure of the QLEDs with the PTAA HTL was investigates using the cross-sectional high-resolution transmission electron microscopy (HR-TEM) images and energy-dispersive spectroscopy (EDS) data obtained using a JEM-2100F (JEOL). The interfacial electronic structures and chemical states were investigated using XPS and UPS (Thermo fisher, NEXSA). Ultraviolet (He I, 21.22 eV) and X-ray (Al Kα, 1486.8 eV) was used as a photon sources for UPS and XPS, respectively. The energy reference of XPS and UPS spectra was calibrated with respect to the Fermi level (E_F_) of clean Au sample. The absorption spectra were measured using UV–vis spectroscopy (JASCO). The CA were measured using a Phoenix 300 (SEO). The electroluminescence characteristics of the QLEDs were measured using a conventional measurement system (M6100, McScience). The optical properties of the PDs were characterized using a probe station and semiconductor parameter analyzer (HP 4145 B). Lasers (CPS635, CPS520, CPS450 and CPS405-Thorlabs) with wavelengths of 635, 520, 450 and 405 nm were used for photosensitivity measurements and photodetector calculations. The P of the laser was measured using a photodetector (S121C-Thorlabs).

## Supplementary Information

Supplementary Information.

## Data Availability

The datasets used and/or analyzed during the current study available from the corresponding author on reasonable request.
