# Supplementary material for: Enhanced performance of flexible quantum dot light-emitting diodes using a low-temperature processed PTAA hole transport layer
Source: Sci Rep. 2023 Mar 7;13:3780. doi: 10.1038/s41598-023-30428-y (PMC9992373; doi:10.1038/s41598-023-30428-y)
Supplement: Supplementary file 1 — Supplementary Information. [file 41598_2023_30428_MOESM1_ESM.pdf]

SUPPORTING INFORMATION

Enhanced performance of flexible quantum dot light-emitting diodes using a low-temperature processed PTAA hole transport layer

Hyoun Ji Ha[1,2], Min Gye Kim[1,2], Jin Hyun Ma[1,2], Jun Hyung Jeong[1,2] , Min Ho Park[1,2], Seong Jae Kang[1,2], Wonsik Kim[3], Soohyung Park[3,4], Seong Jun Kang[1,2,]*

[1] *Department of Advanced Materials Engineering for Information and Electronics, Kyung Hee University, Yongin 17101, Republic of Korea*

[2] *Integrated Education Program for Frontier Materials (BK21 Four), Kyung Hee University, Yongin 17104, Republic of Korea*

[3] *Advanced Analysis Center, Korea Institute of Science and Technology, 5 Hwarang-ro 14-gil, Seongbuk-gu, Seoul 02792, Republic of Korea*

[4] *Division of Nano & Information Technology, KIST School, University of Science and Technology (UST), Seoul 02792, Republic of Korea*

Index

Figure S1. EDS mapping images of QLEDs with UVO 40

Figure S2. J-V curves of the HOD with pristine, UVO 30, UVO 40 and UVO 60.

Figure S3. (a) O1s XPS spectra of ITO and N1s spectra of PTAA film on ITO (b) pristine (c) UVO 40

Figure S4. CA images with (a) pristine (b) UVO 30 (c) UVO 40 (d) UVO 60

Figure S5. Tauc's plots of the absorbance spectra (a) V_2O_5 (b) TFB (c) PTAA (d) QDs

Figure S6. J-V curves of the QLEDs with TFB and UVO 40 HTL

Table S1. Device performance of the QLEDs on the ITO glass substrates with various concentration of PTAA HTLs.

Table S2. Device performance of the QLEDs on the ITO glass substrates with various UVO treatment time on the PTAA surface.

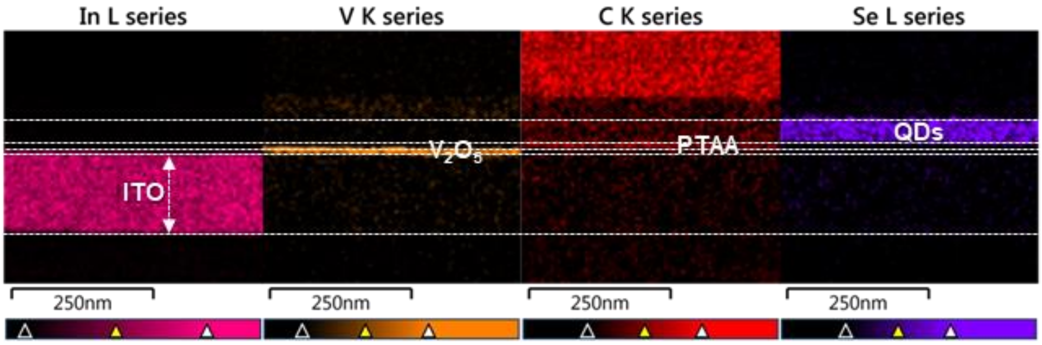

Figure S1. EDS mapping images of QLEDs with UVO 40

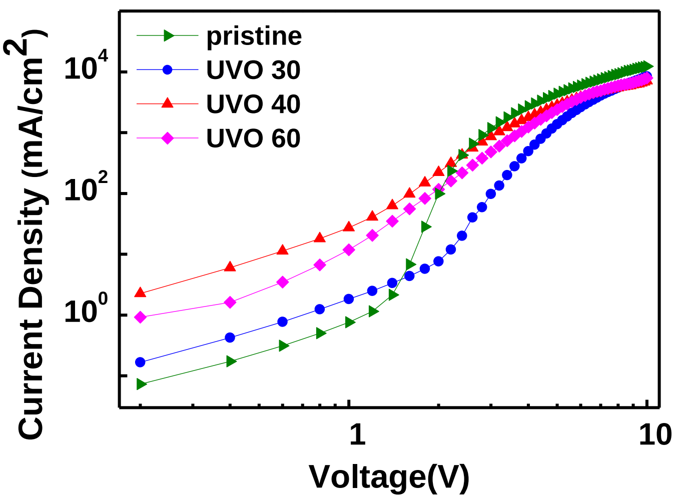

Figure S2. J-V curves of the HOD with pristine, UVO 30, UVO 40 and UVO 60.

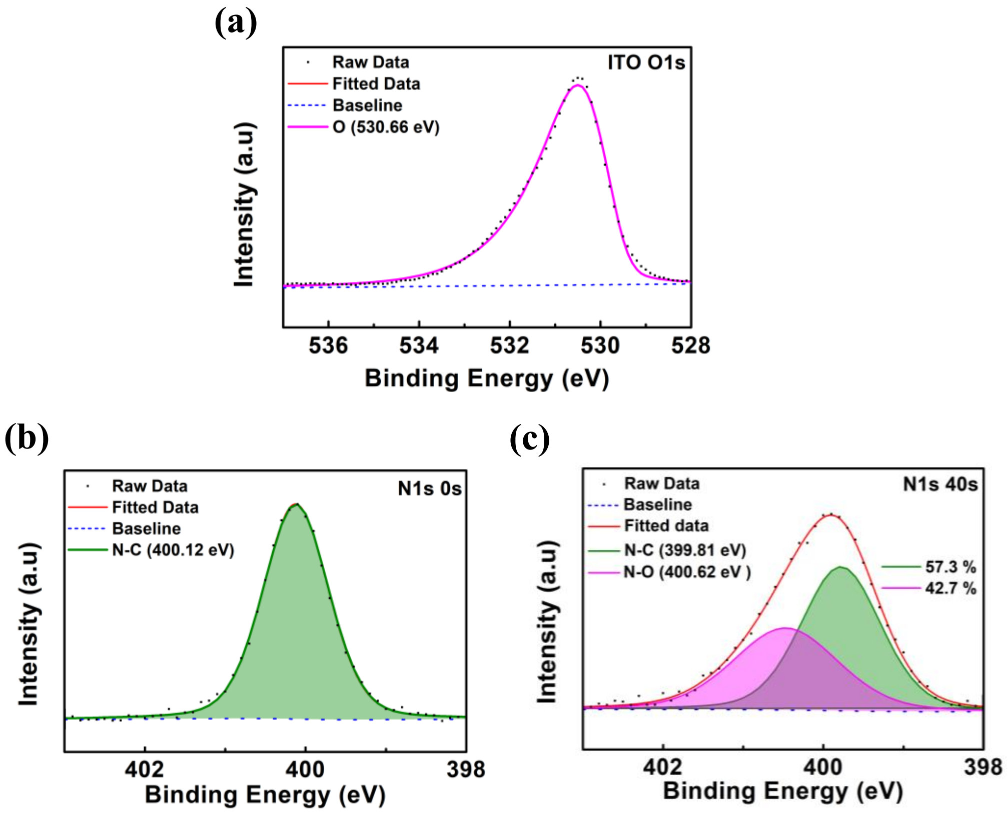

Figure S3. (a) O1s XPS spectra of ITO and N1s spectra of PTAA film on ITO (b) pristine (c) UVO 40

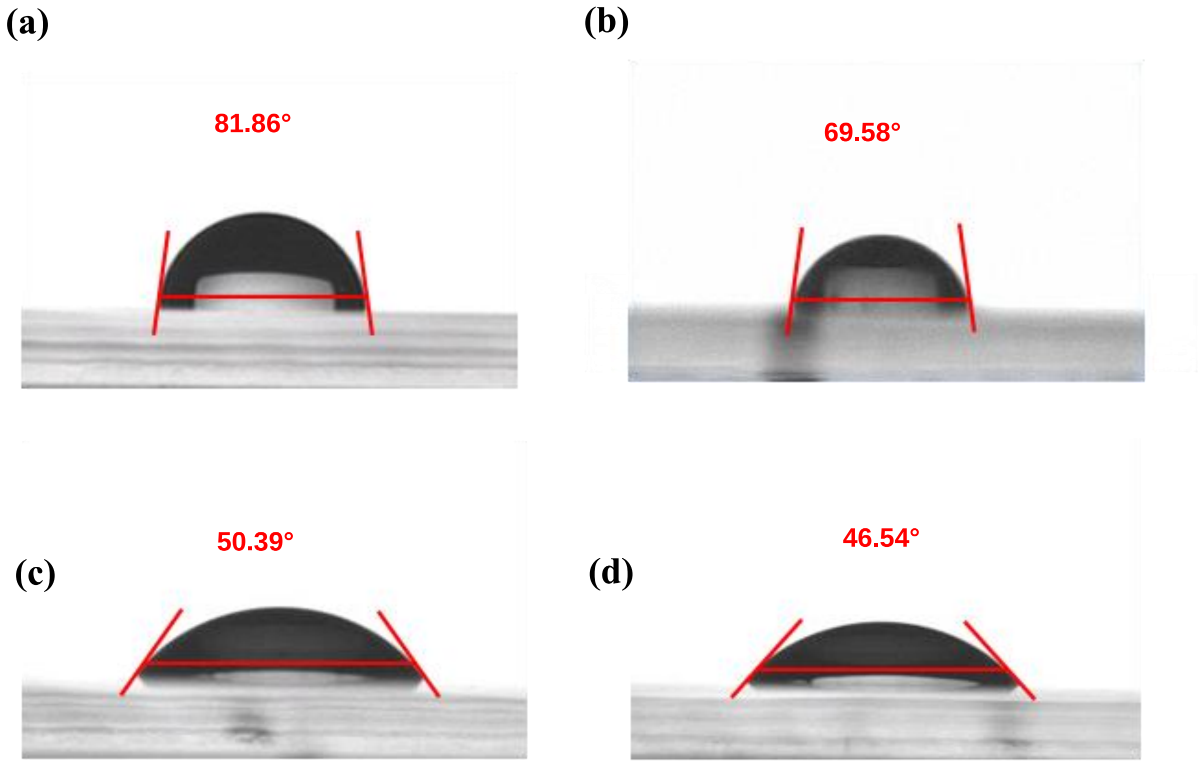

Figure S4. CA images with (a) pristine (b) UVO 30 (c) UVO 40 (d) UVO 60

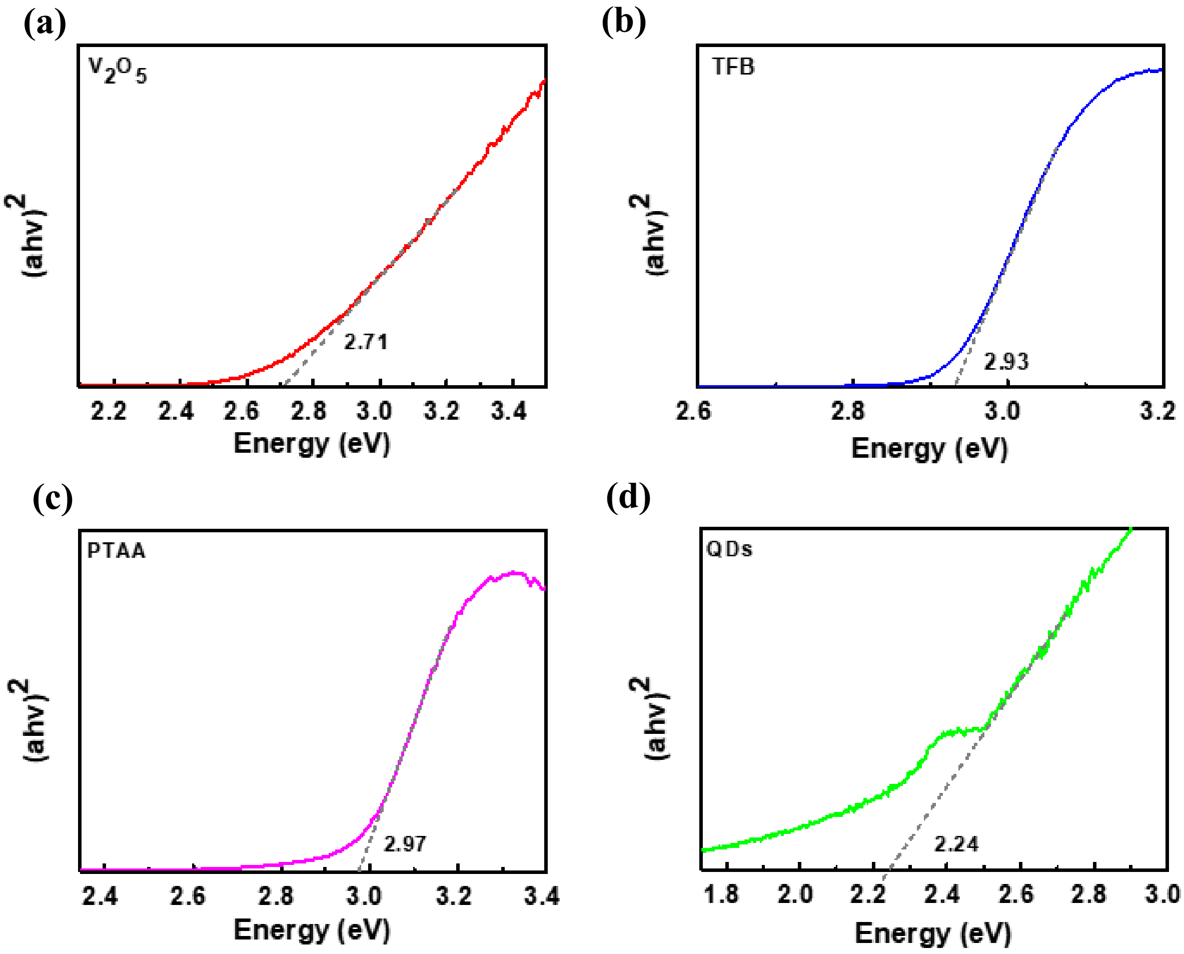

Figure S5. J-V characteristics of (a) QLEDs with ATO 180, ATO 120, ATO 60, and without any interfacial layer and (b) the analysis of ATO 120 using SCLC theory.

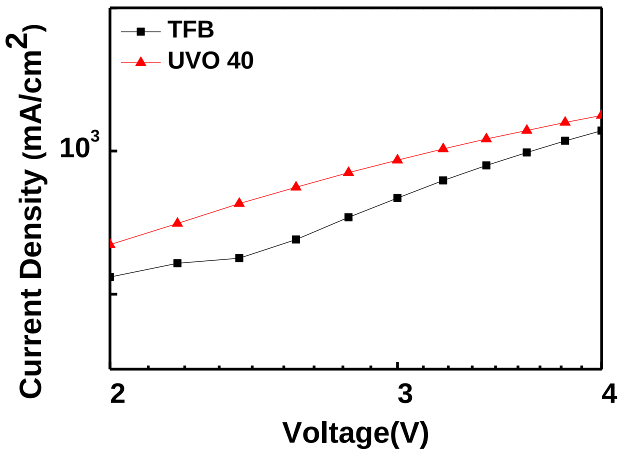

Figure S6. J-V curves of the QLEDs with TFB and UVO 40 HTL

Device	L_{max} (Cd/m^2)	Turn on V (V)	CE_{max} (Cd/A)	PE_{max} (Im/W)	EQE (%)	Full width half maximum (nm)	EL λ_{max} (nm)
2 mg/ml	2.1×10^4	2.9	5.1	2.5	1.1	23.6	535
4 mg/ml	8.5×10^4	2.9	8.8	3.6	1.9	24.9	538
6 mg/ml	8.9×10^4	2.7	15.9	7.3	3.5	24.6	535
8 mg/ml	6.7×10^4	2.7	8.5	4.0	1.9	24.8	537
10 mg/ml	1.6×10^4	3.0	2.5	1.0	0.6	25.8	535

Table S1. Device performance of the QLEDs on the ITO glass substrates with various concentration of PTAA HTLs.

**Device**	**L_{max} (Cd/m^2)**	**Turn on V (V)**	**CE_{max} (Cd/A)**	**PE_{max} (Im/W)**	**EQE (%)**	**Full width half maximum (nm)**	**EL λ_{max} (nm)**
PTAA pristine	2.9×10^4	2.3	2.2	1.4	0.5	24.1	537
UVO 30	4.3×10^4	3.3	6.4	2.5	1.5	23.9	532
UVO 40	8.4×10^4	3.3	11.6	4.9	2.7	24.1	533
UVO 60	6.1×10^4	3.3	8.3	3.2	1.9	24.2	533
UVO 180	1.7×10^4	3.5	2.7	1.1	0.6	24.7	532

Table S2. Device performance of the QLEDs on the ITO glass substrates with various UVO treatment time on the PTAA surface.
